# Can electrophysiological information reflect the response of mangrove species to salt stress? A case study of rewatering and Sodium nitroprusside application

**DOI:** 10.1080/15592324.2022.2073420

**Published:** 2022-05-18

**Authors:** Kashif Ali Solangi, Yanyou Wu, Deke Xing, Waqar Ahmed Qureshi, Mazhar Hussain Tunio, Sher Ali Sheikh, Abdul Shabbir

**Affiliations:** a Key Laboratory of Modern Agricultural Equipment and Technology, Ministry of Education, Institute of Agricultural Engineering, Jiangsu University, Zhenjiang, Jiangsu, China; b and Technology, State Key Laboratory of Environmental Geochemistry, Chinese Academy of Sciences, Institute of GeochemistryResearch Centre for Environmental Bio-Science, Guiyang, Guizhou, China; c Research Centre of Fluid Machinery Engineering and Technology, Jiangsu University, Zhenjiang, Jiangsu, China

**Keywords:** *Aegiceras corniculatum*, bioenergetics, *Kandelia obovate*, total salt resistance capacity, ultrafiltration capacity

## Abstract

The changes in plant life behaviors and water status are accompanied by electrophysiological activities. In this study, the theoretical relationship between clamping force (C_F_) and leaf resistance (R), capacitive reactance (X_C_), inductive reactance (XL), impedance (Z), and capacitance (C) were exposed as 3-parameter exponential decay and linear models based on bioenergetics, respectively, for mangrove species. The intracellular water metabolism parameters and salt transport characteristics were also determined based on mechanical equations with influences of Sodium nitroprusside (SNP) and rewatering (R_W_). The results show that the inherent capacitance and effective thickness could better represent *Aegiceras corniculatum* (*A. corniculatum*) species, and inherent resistance and impedance show obvious effects on *Kandelia obovate* (*K. obovate*) species at different salt levels. SNP application shows positive effect on different salt-resistance capacities of *A. corniculatum*, while *K. obovate* perform better in R_W_ phase at high salt level. These outcomes indicates that *K. obovate* is more salt-resistant because R_W_ process is consistent with actual situation, and response of *A. corniculatum* at high salt stress is irreversible, even in R_W_. It is concluded that the electrophysiological parameters could be used for the determination of salt-resistant capacities, which gave more enhanced and reliable information of mangroves’ life activities.

## 1. Introduction

The life activities of plant involve salt tolerant capacity, water status, stress resistance, metabolism of substance and energy, growth, and other processes.^1,2^ Various changes in plant physiological processes such as photosynthesis, stomatal conductance, respiration, transpiration, substance flow, energy metabolism, and plant growth are directly or indirectly linked with electrical signal (variation, action, and system potential).^3^ The relationship between electrical and physiological processes in plant can be analyzed by the simulation.^4^ The membrane lipid is an important element of the cell membrane, and it could be acting as an insulating layer along with electrical resistance, which allows the plant cell to store the electric charge.^5^ Moreover, through transpiration, approximately 95% of water in the plant leaves dissipate, and remaining 5% of water in the leaf cell are available to support their growth.^2^ This 5% precious intracellular water is utilized for the metabolism, which performs an important role in the physiological processes.^6^ Plant cells injured by abiotic or biotic stress at that time produce variations in structure and composition ion permeability, and consequential changes were observed in the electrical parameters.^7^ These variations are considered as a quick response when plants are under stressful environment.^8^ Hence, the electrical parameters can be clearly influenced by environmental changes and other aspects. Consequently, a method was developed on an urgent basis to monitor the electric parameters of mangrove species with high reproducibility.

Mangrove plays an important role in controlling the soil salinity in coastal areas; it decreases the sea intrusion and increases the irrigated land.^9^ Mangrove forest provides a vast range of economic and ecological benefits.^10^ Mangrove species are divided into two categories based on their special features, such as true mangrove and associate mangrove. *Aegiceras corniculatum* (*A. corniculatum*) is a salt-secretor mangrove, and *Kandelia obovate* (*K. obovate*) is a non-secretor mangrove, but both are the true mangrove species.^11^ *A. corniculatum* has salt glands in their leaves and thus are considered as a salt-secretor mangrove; the salt glands are important strategy to sustain osmotic balance and increase salt-tolerance rate.^12^ *K. obovate* has evolved many mechanisms to resist high salinity, and the most important feature is the maintenance of cell turgor by accumulating inorganic ions in the vacuole and organic compounds in the cytoplasm.^13^ Generally, plant cell performs the important role and involves in different salt-tolerant mechanism, such as salt accumulation of plastoglobuli by *K. obovate* and secretion by *A. corniculatum* are important features in opposition to the salt stress.^14^

How electrophysiological information reflect the mangrove plants in stress condition? Could electrophysiological parameters describe the inherent relationship between electrical parameters and stressful environment? The physiological capacitance and impedance are always used for the evaluation of water status and plant growth.^15^ Ion groups of mesophyll cell are the electrolytes, which are strongly associated with the electrical processes. During the stress condition, salt enters the plant cell and vacuoles, and the vacuoles perform the main role of salt storage capacity. Additionally, the electrical parameters are connected with the plant cell and vacuole volume. Therefore, leaf capacitance (C) performs the main role during the determination of water status, and C is associated with the effective thickness and the area of the leaves in contact with the capacitor plates.^16^ In the present study, C was measured with the help of clamping force (C_F_); in this method, the leaf was clipped between two electrodes of the parallel plate capacitor, and the detailed descriptions of parallel plate capacitor sensor are shown in Figure 3. The inherent relationship between C_F_ and R, X_C_, XL, Z, and C of mangrove species has not yet been revealed. Therefore, it is an effective approach to expose the inherent relationship between plant electrical parameters and C_F_ to provide efficient technique for monitoring physiological condition of mangroves.

The metabolism water that is occupied in leaf cell impact the concentration of electrolytes (electric dipoles and ions groups) and, thus, attend the dynamic electrical activity. The leaf capacitance (C), resistance (R), and impedance (Z) related to intracellular water metabolism, electric dipoles, ions, and ion groups resulted in variations in the electrolyte concentration. The physical models and inherent mechanisms involved between C_F_ and R, X_C_, XL, Z, and C values of mangrove leaves were revealed in this study. Furthermore, from the leaf C, X_C_ values can be calculated. Subsequently, the inherent resistance (IR), capacitive reactance (IX_C_), inductive reactance (IXL), the intrinsic impedance (IZ), capacitance (IC), and specific effective thickness (ET) of the inherent electrical parameters in the plant leaves were successfully obtained using the respective mechanism equations. The intracellular water metabolism parameters such as water-holding capacity (IWHC), water-use efficiency (IWUE), and water-holding time (IWHT) were defined based on the inherent electrical parameter equations and reflect the intracellular water metabolism.

Moreover, the cell membrane performed a vital role to stabilize the environment inside the cell. The energy used by cells in salt transport is almost 60% of the total energy consumed by the cells.^7^ The type of surface and binding ability of salt in cell membrane is most closely related with salt transport capacity; therefore, salt transport capacity of cells is reflected by the composition and content of the membrane protein. Therefore, in this study, salt transport capacity of mangrove leaves were investigated by their mechanism equations with the help of inherent electrical parameter. The salt flux per unit area (USF), salt transfer rate (STR), and salt transport capacity (STC) in plant leaves in the light of the inherent electrophysiological parameters were defined to assess the salt transport strategies. Moreover, salt uptake helps to maintain positive pressure potential through their contribution to osmotic adjustment of growing tissues.^17^ However, under high salinity conditions, the survival of the plant depends on its ability to regulate the internal salt concentrations and prevent ions from reaching toxic levels.^18,19^

Generally, salt-resistant capacity of mangrove species depends on its cell volume, which is connected with salt outflow capacity, which is determined by inherent electrical parameters such as IR, IX_C_, IXL,and IZ. Furthermore, as we know that mangroves are considered salt-resistant species; therefore, in high salt level, mangroves upstand with their unique features. Salt dilution capacity (large capacity) of mangrove species can be determined by inherent electrical parameters such as CP, ET, IWHC, and STC. Moreover, mangroves develop different mechanism associated with anatomic and physiological characteristics to regulate salt absorption and exclusion, such as salt ultrafiltration and salt secretion. Salt ultrafiltration capacity is also an important factor, in which the mangroves filter the salt with their root and leave. Therefore, in this study, salt ultrafiltration capacity is also determined by the electrical parameters such as IWUE, IWHT, USF, and STR.

Furthermore, the high concentration of Na^+^ in the soil solution, beyond the normal condition, decreased the water potential at certain levels. Hence, the plant roots will not able to take up enough water for metabolism, and so the plant suffers physiological drought (Xing et al., 2019). Sodium nitroprusside (SNP) is a crucial signaling molecule that performs important role in various physiological processes, such as senescence and adaptive responses to water deficit and salinity stresses.^20,21^ Jian et al. (2016) suggested that the exogenous SNP could alleviate the damage caused by various abiotic stress. Various studies reported that the application of low concentration of SNP could improve the salinity-induced oxidative injury in the roots of cucumber,^22^ seed of alfalfa,^23^ and cotton seedling.^24^ Furthermore, SNP could increase chlorophyll content and maintain the stability of photosynthetic complexes in thylakoid membranes, which was helpful to plants, especially under stress conditions.^25,26^ So, this study also evaluates the influence of SNP application on both mangrove species in different salt levels.

The rewatering (R_W_) technique could be a supportive technique, which helps to maintain plant growth by gradually decreasing salt concentration in saline water. In previous study, the rewatering technique used^27^ predict that the rewatering time point could be attained through online monitoring of electrophysiological parameters such as leaf tensity. But this study used the R_W_ technique, which is considered as an intertidal condition for mangroves. Furthermore, the physiological parameters such as net photosynthesis rate (P_N_), leaf stomatal conductance (gs), leaf intercellular CO_2_ concentration (Ci), and transpiration rate (Tr,) were also investigated, which were considered as sensitive parameters in stress conditions. Photosynthetic parameters are continuously inhibited when the plant suffered high stressful environmental conditions.^28^ Therefore, in this study, the photosynthetic parameters considered are used to verify the electrophysiological parameters because electrophysiological parameters can reflect the salt-resistant capacity and drought resistant even when the plant is in healthy condition.

It was noticed that there was not any available method to characterize the difference between different plant species based on salt outflow capacity (C1), salt dilution capacity (C2), and ultrafiltration capacity (C3), neither the common photosynthetic parameter. The electrophysiological parameters may have the potential to characterize the differential response of different plants with these three capacities. Thus, the purpose of this study was to explore the salt-resistant capacities of two mangroves species based on electrophysiological parameter with the help of R_W_ and SNP application and evaluate the status of water metabolism with the help of IWUE and IWHT and also monitor the plant salt transport parameter (USF, STR, and STC) strategies by mechanism equations of both mangrove species in Ss and R_W_ conditions. Furthermore, in this study, the photosynthesis parameters and water potential were also determined to verify the electrophysiological parameters. Moreover, based on the electrophysiological parameters, the C1, C2, and C3 capacities were also calculated because these capacities accurately address salt-resistant capacity of both mangrove species and different adaptive strategies under different salt levels.

## 2. Materials and method

### 2.1. Experimental design and salt treatments and rewatering

**Figure 1.**
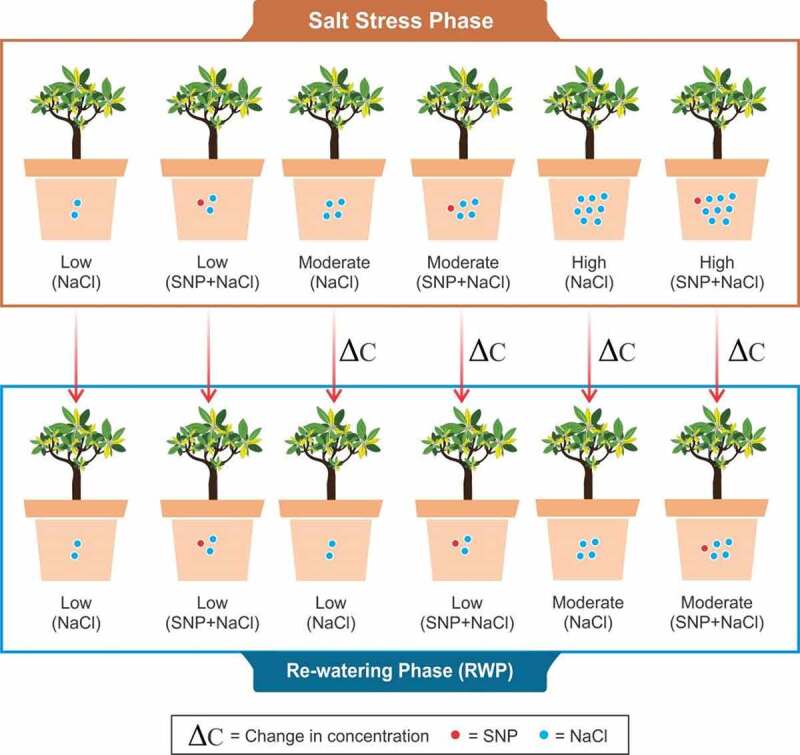
Experiment design shows two different phases (salt stress and rewatering). ΔC indicates the change in NaCl concentration from high to low.

The experiment was performed in a greenhouse of Jiangsu University, Zhenjiang, Jiangsu, China, from 11 September to 11 November 2019. Two different mangrove species were collected from Quanzhou Tongqing mangroves technology Co. Ltd., Fujian, China. Initially, both the species were washed with tape water and then stored in 10 liter of half strength nutrient Hoagland solution.^29^ The youngest leaves of fresh branches were selected as the experimental material taken from the fifth and fourth leaf positions of each branch. Moreover, a complete randomized block design was used with six different salt treatments, and each treatment contains four replicates. The mangrove species were kept for 30 days in salt-stress phase; after that, the same time span was used for the R_W_ phase. In salt-stress phase, different NaCl treatments were used, i.e., T1: NaCl (100 mM) considered as (low NaCl); T2: SNP (0.01 mM) + NaCl (100 mM) (low NaCl + SNP); T3: NaCl (200 mM) (moderate NaCl); T4: SNP (0.01 mM) + NaCl (200 mM) (moderate NaCl +SNP); T5: NaCl (400 mM) (high NaCl); and T6: SNP (0.01 mM) + NaCl (400 mM) (high NaCl + SNP). Regarding R_W_, the phase order was as follows: T1: NaCl (100 mM) considered as (low NaCl); T2: SNP (0.01 mM) + NaCl (100 mM) (low NaCl + SNP); T3: NaCl (100 mM) (low NaCl); T4: SNP (0.01 mM) + NaCl (100 mM) low NaCl + SNP); T5: NaCl (200 mM) (moderate NaCl); and T6: SNP (0.01 mM) + NaCl (200 mM) (moderate NaCl +SNP), as shown in Figure 1.

**Figure 2.**
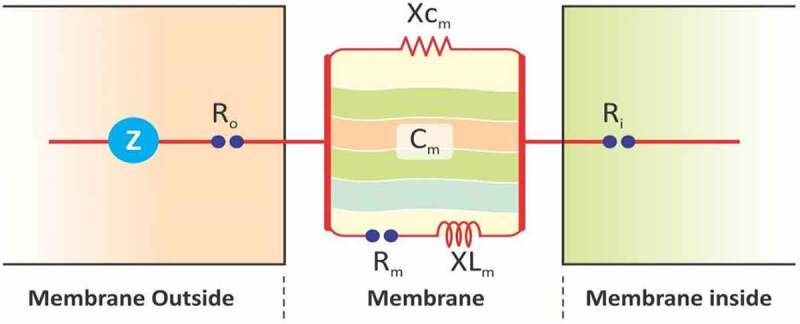
Simplified equivalent circuit of cells. Z = impedance, C_m_ = capacitance of membrane, R_m_ = resistance of membrane, X_Cm_ = capacitive reactance of membrane, XL_m_ = inductive reactance of membrane, Ro = resistance outside membrane, and Ri = resistance inside membrane.

**Figure 3.**
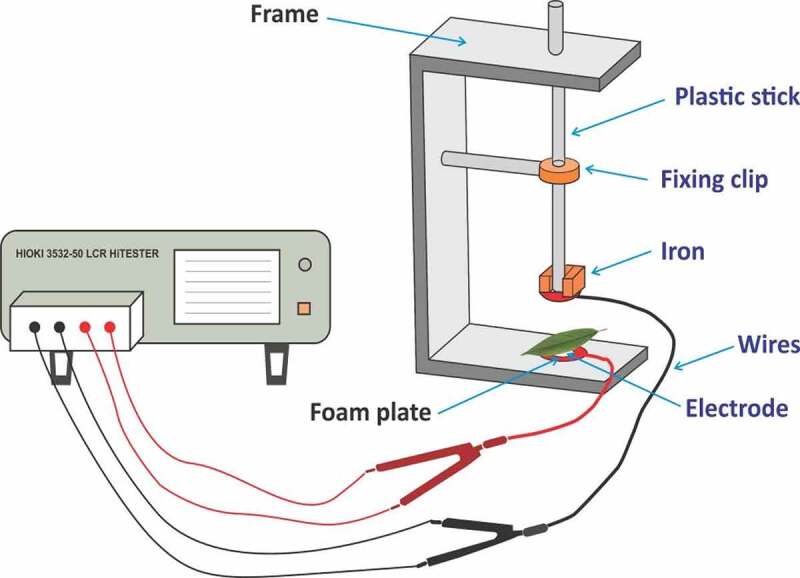
Representation of the parallel plate capacitor.

### 2.2. Inherent mechanism relationships of C_F_ and leaf R, X_C_, and XL

Mesophyll cell is considered as a concentric sphere capacitor with both resistor and inductor functions.^2^ The diagram of a simplified equivalent circuit of mesophyll cell is shown in Figure 2.

The plasmodesmata is linked with mesophyll cells, so it generates low-resistance ways to pass by electrical current. Generally, mesophyll cells can be separated into unreliable spherical tissue cells and long cylindrical palisade tissue. To simplify and achieve this scientific issue, every mesophyll cell can be observed with a concentric sphere capacitor. Therefore, the leaf capacitor consisted of aligned mesophyll cell. The values of Z, R, and C are based on the electrolyte concentration in the leaf; therefore, the parallel connection mode of LCR was used. The water status of the leaf cells depends on the variation in the electrolyte concentration.^30^ The above-mentioned parameters (Z, R, and C) were evaluated by LCR tester (3532–50, HIOKI, Japan), as shown in Figure 3.

The voltage and frequency used were 1 V and 3 kHz, respectively. A leaf was placed between two parallel electrode plates, and a clamping force (C_F_) was applied. After that sigma plot (version 12.0) was used and applied to polynomial linear equation for curve fitting. Finally, one equation was obtained from each repeat, which is mentioned above in detail, and leaf Z, R, and C at different C_F_ were measured continuously by adding the same quantity and quality of iron block. Finally, the values of leaf X_C_ and XL are expressed as follows:

(1) $$XC=\frac{1}{2\pi fC}$$

(2) $$\frac{1}{-XL}=\frac{1}{Z}-\frac{1}{R}-\frac{1}{Xc}$$

Where X_C_ = capacitive reactance, π = 3.1416, f = frequency, C = physiological capacitance, XL = inductive reactance, Z = Impedance, and R = resistance.

The concentrations of the electrolytes are associate to Z outer and inner side of the cell membrane, which is defined by plant leaf Z. The outside stimulation changes in membrane permeability of electrolytes impact the outer and inner side of cell membrane. According to the bioenergetics, the potential of ion groups and electric dipoles inside and outside the cell membrane can be obtained by Nernst equation.^2^ Thus, the Nernst equation can be obtained from equation 3 as follows:

(3) $$E-E^{0}=\frac{R_{o}T}{n_{z}F_{o}}ln\frac{Qi}{Qo}$$

where E is the electromotive force (V), $E^{0}$ is the standard electromotive force (V), $R_{o}$ is the gas constant (8.314570 J K^−1^ mol^−1^), T is the thermodynamic temperature (K), Qi is the concentration of electrolytes responding to Z inside the cell membrane (mol L^−1^), Qo is the concentration of electrolytes responding to Z outside the cell membrane (mol L^−1^), F_0_ is the Faraday constant (96,485 C mol^−1^), and $n_{z}$ is the number of transferred electrolytes (mol).

Hence, the pressure work applied on leaf cells can be converted into internal energy of electromotive force, and they have a direct relationship, PV = a E:

(4) $$PV=aE=aE^{o}+\frac{aR_{o}T}{n_{z}F_{o}}ln\frac{Qi}{Qo}$$

where P is the pressure intensity on the leaf cells (Pa), a is the energy conversion coefficient of the electromotive force, and V isthe cell volume (m^3^). Further, $P=\frac{F}{S}$, where F is the clamping force (N) and S is the effective area of the electrode plate (m^2^). F can be calculated by the gravity formula:

(5) $$F=\;\left(M+m\right)g$$

where M is the iron block mass (kg), m is the mass of the plastic rod and the plate electrode (kg), and g = 9.8 N/kg.

For mesophyll cells, the sum of Qo and Qi is certain. Qi is directly proportional to the conductivity of electrolytes that respond to Z, and the conductivity is the reciprocal of Z. Hence, $\frac{Q_{i}}{Q_{o}}$ can be expressed as $\frac{Q_{i}}{Q_{o}}=\frac{\frac{J_{o}}{z}}{Q-\frac{J_{o}}{z}}=\frac{J_{o}}{QZ-\;J_{o}}$, where J_O_ is the ratio coefficient of the conversion between Qi and Z and Q is Qo + Qi. Therefore, Formula (2) can be transformed into equation (6)..

(6) $$\frac{V}{S}F=aE^{o}-\frac{aR_{o}T}{n_{z}F_{o}}ln\frac{QZ-\;J_{o}}{J_{o}}$$

(7) $$\frac{aR_{o}T}{n_{z}F_{o}}ln\frac{QZ-\;J_{o}}{J_{o}}=aE^{o}-\frac{V}{S}F$$

and

(8) $$\;\;ln\frac{\;\;QZ-\;{\;\;J}_{\;\;o}}{{\;\;J}_{\;o}}=\frac{{\;n}_{\;z}{\;F}_{\;o}{\;E}_{\;o}}{\;RT}-\frac{\;V{\;n}_{\;Z}{\;F}_{\;o}}{\;SaRT}\;F$$

The exponents of both sides in equation (9):

(9) $$\frac{\;QZ-\;{\;J}_{\;o}}{{\;J}_{\;o}}={\;e}^{\frac{{\;n}_{\;z}{\;F}_{\;o}{\;E}_{\;o}}{{\;R}_{\;o}\;T}}{\;e}^{\left(-\frac{\;V{\;n}_{\;Z}{\;F}_{\;o}}{\;S\;a{\;R}_{\;o}\;T}\;F\right)}$$

Further,

(10) $$\;Z=\frac{{\;J}_{\;o}}{\;Q}+\frac{{\;J}_{\;o}}{\;Q}\;{\;e}^{\frac{{\;n}_{\;z}{\;F}_{\;o}{\;E}_{\;o}}{\;R_{o}\;T}}{\;e}^{\left(-\frac{\;V{\;n}_{\;Z}\;F_{o}}{\;S\;a\;R_{o}T}\;F\right)}$$

As d=vs,Formula(10)istransformedinto

(11) $$\;Z=\frac{{\;J}_{\;o}}{\;Q}+\frac{{\;J}_{\;o}}{\;Q}\;{\;e}^{\frac{{\;n}_{\;Z}\;F_{o}\;E_{o}}{\;R_{o}\;T}}{\;e}^{\left(-\frac{\;d{\;n}_{\;Z}\;F_{o}}{\;a\;R_{o}\;T}\;F\right)}$$

For the same leaf tested in the same environment, the d, a, $E_{o}$, $R_{o}$, T, $n_{z}$, $F_{o}$, Q, and $J_{o}$ of Formula (10) are constants. Let $y_{o}=\frac{J_{o}}{Q},$ $k_{1}=\frac{J_{o}}{Q}$ ${\;e}^{\frac{\;nzF_{o}\;E_{o}}{\;R_{o}\;T}},$ and ${\;b}_{1}=\frac{\;d\;nZFo}{\;a\;R_{o}\;T}$, then the intrinsic mechanical relationships of leaf Z and F are

(12) $$Z=y_{o}+k_{1}e^{-b_{1}F}$$

where y0, k_1_, and b_1_ are the model parameters.

When F = 0, the inherent impedance (IZ) can be determined by

(13) $$IZ=y_{o}+k_{1}$$

same as the Z, and the inherent mechanical relationships of leaf X_C_ and F are revealed.

(14) $$Xc=p_{o}+k_{2}e^{-b_{2}F}$$

where $p_{o}$, $k_{2}$, and $b_{2}$ are the model parameters. When F = 0, the intrinsic capacitive reactance (IX_C_) (IR) of the plant leaves can be calculated as

(15) $$IXc=p_{o}+k_{2}$$

Like R, the inherent mechanism relation of leaf XL and F are calculated as

(16) $$XL=q_{o}+k_{3}e^{-b_{3}F}$$

When F = 0, the inherent inductive reactance (IXL) of the plant leaves could be calculated as

(17) $$IXL=q_{o}+k_{3}$$

Thus, the inherent impedance (IZ) and inherent capacitance (IC) of the plant leave were, respectively, obtained according to equations (18) and (19)

(18) $$\frac{1}{-XL}=\frac{1}{Z}-\frac{1}{R}-\frac{1}{Xc}$$

The clamping force according to the first law of thermodynamics were obtained as follows

(19) $$IC=\frac{1}{2\pi fIXc}$$

The Gibbs free energy equation is

(20) ΔG=ΔH+PV

where ΔG is Gibbs free energy, ΔH is the internal energy of the leaf cell system, P is the pressure intensity of the leaf cells, and V is the cell volume (m^3^). P can be calculated by the pressure intensity equation:

(21) P = F/A

where F is the clamping force (N) and A is the effective area of the electrode plate (m^2^).

The mesophyll cells could be regarded as concentric sphere capacitors. The equation for the energy of spherical capacitor is

(22) $$W_{C}=\;1/2\;U^{2}CP$$

where W_C_ is the capacitor energy, U is the test voltage (V), and C is the physiological capacitance (pF). According to energy conservation theory, a capacitor’s energy is equal to the work converted by the Gibbs free energy, i.e., W = ΔG. The leaf C and clamping force (C_F_) relationship model can thus be obtained by

(23) $$\;C=\frac{2\;\Delta H}{{\;U}^{2}}+\frac{2\;V}{\;A{\;U}^{2}}\;CF$$

By assuming that ET is the specific effective thickness of the plant leaves, therefore, ET = V/A; equation (24) can be rewritten as

(24) $$\;C=\frac{2\;\Delta H}{{\;U}^{2}}+\frac{2\;ET}{{\;U}^{2}}\;CF$$

We assume that $b_{0}=\frac{2\Delta H}{U^{2}},K=\frac{2ET}{U^{2}}$, then equation (25) can be transferred as

(25) $$C=b_{0}+KF$$

Equation (25) is a linear model equation, where

C is thecapacitance (pf), b_0_ and K are the model parameters, and C_F_  is theclamping force (N).

As K = $\frac{2ET}{U^{2}}$, the specific effective thickness (ET) of the plant leaves can be calculated as

(26) $$ET=\frac{U^{2}K}{2}$$

### 2.3. Intracellular water utilization parameters

The cell is of sphere shape, and increase in volume of the cell is associate with their growth. The concentric spherical capacitor formula helps to calculate the C value of the plant leave.

(27) $$Cc=\;\frac{4\pi \varepsilon R1R2}{R1-R2}$$

where π = 3.1416, Cc is the capacitance of the concentric spherical capacitor (pF), ε is the dielectric constant of electrolytes, R_1_ is the outer sphere radius (m), and R_2_ is the inner sphere radius (m). For a plant cell, R_2_ − R_1_ is the thickness of the cell membrane. R_1_ ≈ R_2_, ε, and the thickness of the cell membrane are constant. Therefore, the cell volume (V_C_) has the following relationship with the cell C

(28) $$VC=\alpha \sqrt{C^{3}}$$

The vacuole volume is directly proportion to cell volume, and the water is an important component of the vacuole and cytoplasm. Furthermore, the water-holding capacity of the cells is directly proportional to $\sqrt{C^{3}}.$ Therefore, $\sqrt{C^{3}}$ can represent the water-holding capacity of the plant leaves. The intracellular water-holding capacity (IWHC) of the plant leaves can be calculated by equation (29) as follows:

(29) $$IWHC=\sqrt{{(IC)}^{3}}$$

ET is the specific effective thickness of leaves that characterizes the growth of the cells. Water-holding capacity provides main support for the cell growth. Hence, the intracellular water-use efficiency (IWUE) of leaves is represented by Formula (30):

(30) $$IWUC=\frac{ET}{IWHC}$$

According to Ohm’s law, IZ: U/Z, where IZ is the physiological current (A), U is the test voltage (V), and Z is the physiological impedance (Ω). At the same time, the current is equal to the product of the capacitance and the differential of voltage in time, as shown in equation (31):

(31) IZ = IC × ∫du

Subsequently, for the essential transformation, the current time is the product of the capacitance and impedance. Therefore, the intracellular water-holding time (IWHT) of plant leaves is characterized as follows (32):

(32) IWHT = IC × IZ

### 2.4. Determination of salt transport parameters

Plant cells have the electrical properties of low capacitance and high resistance, and it could be assumed that electrical cells were connected in parallel manner, and various aligned mesophyll cells make up the leaf capacitor. The value of IR in plant leaf cells can be measured as follows:

(33) $$\frac{1}{IR}=\frac{1}{IR_{1}}+\frac{1}{IR_{2}}+\frac{1}{IR_{3}}+\frac{1}{IRn}$$

We can assume that the resistance of inside and outside membrane is equal; then, IR₁, IR₂, IR₃, and IRn can represent inherent resistance of each unit cell membrane. Hence, the IR of the plant leaves were obtained as follows:

(34) $$\frac{1}{IR}=\frac{n}{IR_{o}}$$

Further, the resistance of the cell membrane closely related to lipids and proteins, so, n can be denoted as the relative number of lipids and proteins that induce membrane R in plant leaves. Finally, the leaf IX_C_ in plant were measured as follows..

(35) $$\frac{1}{IXc}=\frac{P}{IXc_{o}}$$

As we know that capacitive resistance of cell membrane is closely related to the surface proteins, then IX_C_ or p was considered as the relative amount of surface proteins. Therefore, IX_C_ is inversely proportional to p.

The IXL of plant leaf was obtained as

(36) $$\frac{1}{IXL}=\frac{q}{IXL_{o}}$$

As we know that inductive resistance of cell membrane is closely related to the binding proteins, then IXL or q was considered as the relative number of binding proteins. Therefore, IXL is inversely proportional to q.

The cell membrane proteins are most closely related to the salt transport; thus, the relative salt flux per unit area (USF) could be represented by equation (37):

(37) $$USF=\;\frac{p+q}{n}=\frac{\frac{1}{IXc}+\frac{1}{IXL}}{\frac{1}{IR}}=\frac{IR}{IXc}+\frac{IR}{IXL}$$

Salts are soluble in water, and the water transfer rate and the salt transfer rate (STR) are conceptually similar and assigned the same value; thus, it can be calculated by equation (38):

(38) $$STR=\frac{\sqrt{{(IC)}^{3}}}{IC\;\;XI\;\;Z}$$

Therefore, the salt transport capacity (STC) is USF multiplied by STR:

(39) STC = USF ∗STR

Salt outflow capacity is related to the biological current. The greater the biological current, the greater the ability of Na^+^ outflow of the cation opposite to the electron. Therefore, we define I = V/IR, I = V/IX_C_, I = V/IXL, and I = V /IZ as salt outflow capacity. Here, V represents the voltage applied in determination, and I represents the current flowing through the cell membrane. Therefore, finally, we define 1/IR, 1/IX_C_, 1/IXL, and 1/IZ as the portion of salt outflow capacity and normalize the value of 1/IR, 1/IX_C_, 1/IXL, and 1/IZ, respectively, as (0,1) into IR_N_, IXC_N_, IXL_N_, and IZ_N_. If IR_N_, IXC_N_, IXL_N_, and IZ_N_ have the same weight, we define salt outflow capacity as follows:

(40) $$C1\;=0.25\;\;(IR_{N}+IXC_{N}+IXL_{N}+\;IZ_{N})$$

Here C1 is equal to the salt outflow capacity

The cell volume and intracellular salt-holding capacity can be determine by IC, ET, IWHC, and STC. Therefore, we define them as the portion of salt dilution capacity and normalize the value of IC, ET, IWHC, and STC, respectively, as (0,1) into IC_N_, ET_N_, IWHC_N_, and STC_N_. If IC_N_, ET_N_, IWHC_N_, and STC_N_ have the same weight, we define salt dilution capacity (large capacity (C2)) as follows:

(41) $$C2\;=0.25\;(IC_{N}+ET_{N}+IWHC_{N}+STC_{N})$$

The ultrafiltration capacity is related to salt flow rate and flow time through the cell membrane. 1/IWUE represents the salt solution flux, IWHT represents the salt flow time, 1/USF represents the solute flux, and STR represents the flow rate. Therefore, we define 1/IWUE, IWHT, 1/USF, and STR as the portion of ultrafiltration capacity and normalize the value of 1/IWUE, IWHT, 1/USF, and STR, respectively, as (0,1) into IWUE_N_, IWHT_N_, USF_N_, and STR_N_. Assume that IWUE_N_, IWHT_N_, USF_N_, and STR_N_ have the same weight, we define ultrafiltration capacity (C3) as follows:

(42) $$C3\;=0.25\;(IWUE_{N}+IWHT_{N}+USF_{N}+STR_{N})$$

If C1, C2, and C3 have the same weight, we define the total salt resistance capacity (T) as follows:

(43) $$\;T=\left(C1+C2+C3\right)/3$$

### 2.5. Leaf water potential (Ψ)

Leaf water potential (Ψ) indicates whole plant water status, and high leaf Ψ is found to be associate with dehydration avoidance mechanisms. Leaf Ψ is connected with opening of stomata because water moves from root to leaf through the series ‘pipes’ known as xylem and is evaporated through small openings on the leaf surface called stomata. In this study, leaf Ψ is determined by a dew point microvolt meter in a C-52-SF universal sample room (Psypro, Wescor, USA); the measuring time was 9:00–11:00 am in both the phases (S_S_ phase and R_W_ phase).

### 2.6. Photosynthetic traits

The net photosynthesis rate (P_N_), leaf stomatal conductance (gs,) leaf intercellular CO_2_ concentration (Ci), and transpiration rate (Tr,) were recorded using a portable LI-6400XT photosynthesis measurement system (LI-COR, Lincoln, NE, USA). The leaf of both species was enclosed within the chamber and stirred continuously with two fans inside. For each parameters, five repeats were taken; totally, four plants were measured in each treatment. The top of the expanded leaf was used for these measurements during full sunshine.^31^ Throughout the measurement, the set values are as follows: the flow rate inside the chamber, 500 (µmol s^−1^), atmospheric pressure 99.9 kPa, using its own blue and red light, and photosynthetic active radiation (PAR) was 800 (µmol m^−2^ s^1^).

### 2.7. Statistical analysis

he measurements were analyzed using analysis of variance (ANOVA) on SPSS software (version 20.0, SPSS Inc.) to assess the significant modification between diverse salt levels using Duncan’s LSD post hoc test at *p* < .05; The data were shown as the mean ± standard error (SE). The figures of physiological and growth parameters were prepared by Origin Pro. 9.0 (Northampton, MA, USA).

## 3. Results

### 3.1. Confirmation of inherent mechanism relationship

**Figure 4.**
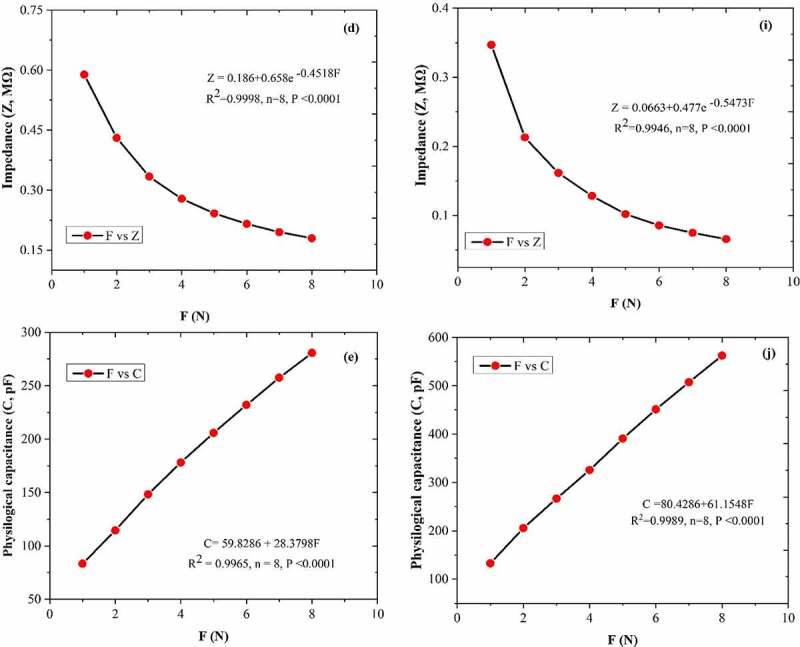
Continued.

Between C_F_ and leaf R, X_C_, XL, Z and C was generated the fitting curves equations; R (Figure 4a), X_C_ (Figure 4b), XL (Figure 4c), Z (Figure 4d), and C (Figure 4e) of the *A. corniculatum* species, while R (Figure 4f), X_C_ (Figure 4g), XL (Figure 4h), Z (Figure 4i), and C (Figure 4j) of the *K. obovate* species were choose randomly from the list of stress phase. The results of fitting curve equations show that the values of R, X_C_, XL, Z, and C correlated with C_F_. The correlation coefficient (R^2^) of both mangrove species was 0.99, and P value was ≤0.0001. These results demonstrate that the C_F_ and leaf R, X_C_, XL, Z, and C show positive correlations and highlight that the inherent relationships of these mechanism are authentic existence.
**Figure 4.**
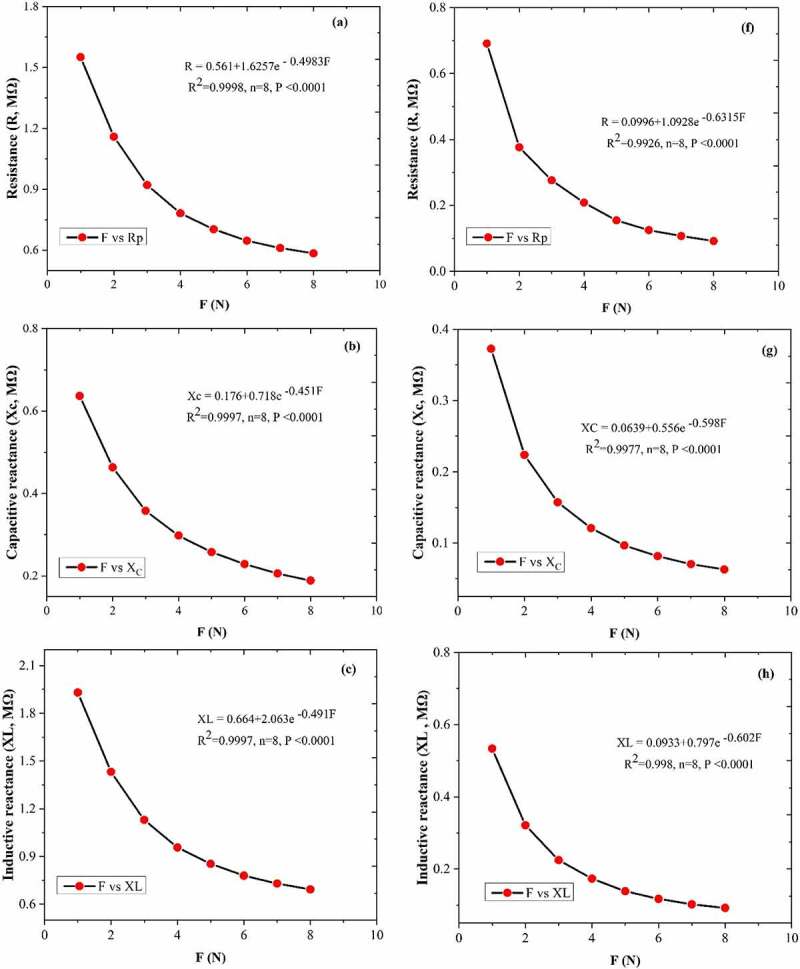
The equations of fitting curve relationship between clamping force C_F_ and leaf resistance (R), capacitive reactance (X_C_), inductive reactance (XL), impedance (Z), andphysiological capacitance (C). Figure (a–e) represents the *A. corniculatum* species and figure (f–j) represents the *K. obovate* species fitting equations in stress condition (random examples).

### 3.2. *Electrophysiological parameter of* A. corniculatum *species*

The results demonstrate that the inherent relationship between C_F_ and leaf R, X_C_, XL, Z, and C of both mangrove species are shown in Figure 4, while inherent electrical parameters including IR, IX_C_, IXL, IZ, IC, and ET of *A. corniculatum* species in S_S_ and R_W_ phase are shown in Table 1. In stress phase, *A. corniculatum* has nonsignificant results, which were observed of IR, IX_C_, and IXL in low and medium salt level, but at high salt levels, the values were increasing. IC results were significantly change higher, values were recorded at low salt level and lower values were at high salt level but ET values nonsignificant and sharply decreased at higher salt level. The SNP application exhibited obvious effects, which could indicate that changes in IX_C_ and IC in different salt levels of other parameters show nonsignificant effects with SNP application in S_S_ phase. Furthermore, it was noted that SNP can be reflected on IX_C_, IZ, and IC in a long-term and low salt environment, while ET increased in medium salt environment. Moreover, rewatering technique does not improve these parameters expected ET. In moderate -low (M-L) and high-medium (H-M) level ET results noticeable changes were seen.

**Table 1.** The electrophysiological parameters of *A. corniculatum* species in salt stress and rewatering phase, clamping force C_F_ = 0

Salt concentrations	Low (NaCl)	Low (SNP +NaCl)	Medium/medium-low (NaCl)	Medium/medium-low (SNP + NaCl)	High/high-medium (NaCl)	High/high-medium (SNP +NaCl)
IR-Ss	4.04 ± 0.73^b^	2.32 ± 0.19^b^	5.52 ± 0.55^b^	3.67 ± 0.62^b^	12.70 ± 7.32^a^	7.49 ± 2.83^b^
IR-R_W_	1.97 ± 0.49^b^	1.74 ± 0.48^b^	5.81 ± 0.78^b^	2.12 ± 0.29^b^	13.2 ± 8.59^a^	9.87 ± 2.26^b^
IX_**C**_-Ss	1.62 ± .08^ab^	1.04 ± .78^ab^	1.41 ± .09^ab^	1.24 ± .12^ab^	3.47 ± .32^a^	1.92 ± .06^b^
IX_**C**_-R_W_	1.45 ± 0.32^ab^	0.77 ± 0.46^c^	1.75 ± 0.24^ab^	1.25 ± 0.02^ab^	2.81 ± 0.68^a^	1.83 ± 0.31^ab^
IXL-Ss	4.24 ± 0.17^b^	3.01 ± 0.17^b^	6.10 ± 0.58^b^	4.36 ± 0.68^b^	33.80 ± 7.25^a^	8.56 ± 1.89^b^
IXL-R_W_	2.90 ± 0.68^b^	2.16 ± 0.44^b^	6.63 ± 0.83^b^	2.98 ± 0.28^b^	38.40 ± 23.70^a^	10.50 ± 2.39^b^
IZ-Ss	1.56 ± 0.33^ab^	0.95 ± 0.59^ab^	1.37 ± 0.91^ab^	1.17 ± 0.12^ab^	3.46 ± 0.34^a^	1.86 ± 0.18^b^
IZ-R_W_	1.17 ± 0.27^ab^	0.69 ± 0.58^c^	1.68 ± 0.23^b^	1.06 ± 0.51^ab^	2.80 ± 0.69^a^	1.81 ± 0.31^b^
IC-Ss	32.9 ± 1.78^ cd^	51.3 ± 4.08^b^	38.1 ± 2.62^bcd^	43.6 ± 4.56^bc^	15.5 ± 1.47^ f^	28.1 ± 2.57^def^
IC-R_W_	39.9 ± 7.94^bcd^	69.4 ± 4.42^a^	31.5 ± 3.97^cde^	42.4 ± 0.78^cde^	20.8 ± 4.07^ef^	31.3 ± 6.61^cde^
ET-Ss	30.0 ± 2.7^b^	27.7 ± 6.1^bc^	29.7 ± 1.90^b^	36.7 ± 10.1^b^	12.4 ± 0.53^d^	15.4 ± 1.41^ cd^
ET-R_W_	31.5 ± 4.5^b^	33.7 ± 0.6^b^	56.1 ± 3.2^a^	24.3 ± 1.3^bc^	24.2 ± 1.9^bcd^	27.9 ± 5.1^bc^

IR = inherent resistance, IX_C_ = inherent capacitive reactance, IXL = inherent inductive reactance, IZ = inherent impedance, IC = inherent capacitance, ET = effective thickness, values indicate the mean ± SE, n = 8. The small letter indicates significant difference at (p < 0.05) using Duncan’s multiple range tests.

### 3.3. *Electrophysiological parameter of* K. obovate *species*

The electrical parameters of *K. obovate* species including IR, IX_C_, IXL, IZ, IC, and ET in S_S_ and R_W_ phase are shown in Table 2. In the stress phase, *K. obovate* species has nonsignificant results noted for IR, IX_C_, IXL, and IZ in all salt level, while IC and ET showed significant results in low and medium salt level in S_S_ phase. These results display that IC and ET are clearly reflecting the *K. obovate* species in stress phase. The SNP application improves the IX_C_ and IXL in high salt environment and ET in low and medium salt level. These results display that SNP clearly reflected IX_C_, IXL, and ET in S_S_ phase. Moreover, when decreased the salt concentration in rewatering phase, the IC values were increased at H-M level and ET values at M-L level, and other parameters show nonsignificant results in R_W_ phase.

**Table 2.** The electrophysiological parameters of *K. obovate* species in salt stress and rewatering phase, clamping force C_F_ = 0

Salt concentrations	Low (NaCl)	Low (SNP +NaCl)	Medium/medium-low (NaCl)	Medium/medium-low (SNP + NaCl)	High/high-medium (NaCl)	High/high-medium (SNP +NaCl)
IR-Ss	2.59 ± 0.90^b^	0.74 ± 0.22^b^	0.93 ± 0.18^b^	0.72 ± 0.14^b^	4.47 ± 1.02^a^	3.18 ± 4.75^b^
IR-R_W_	1.08 ± 0.50^b^	0.70 ± 0.18^b^	1.44 ± 0.29^b^	1.21 ± 0.21^b^	0.69 ± 0.21^b^	2.87 ± 1.63^b^
IX_**C**_-Ss	0.89 ± 0.13^b^	0.63 ± 0.22^b^	0.81 ± 0.13^b^	0.86 ± 0.10^b^	1.63 ± 0.32^ab^	1.97 ± 0.73^ab^
IX_**C**_-R_W_	0.63 ± 0.08^b^	0.54 ± 0.05^b^	0.96 ± 0.16^b^	0.60 ± 0.04^b^	0.98 ± 0.21^b^	2.91 ± 1.32^a^
IXL-Ss	3.01 ± 0.93^b^	1.18 ± 0.19^b^	1.5 ± 0.25^b^	1.34 ± 0.96^b^	5.09 ± 1.03^ab^	8.45 ± 1.89^ab^
IXL-R_W_	1.91 ± 0.50^b^	1.06 ± 0.19^b^	1.97 ± 0.37^b^	1.59 ± 0.25^b^	2.16 ± 0.86^b^	10.1 ± 6.98^a^
IZ-Ss	0.85 ± 0.14^bc^	0.45 ± 0.05^c^	0.60 ± 0.10^bc^	0.53 ± 0.05^c^	1.56 ± 0.31^abc^	1.94 ± 0.74^a^
IZ-R_W_	0.50 ± 0.13^c^	0.42 ± 0.05^c^	0.81 ± 0.15^b^	0.53 ± 0.52^c^	0.50 ± 0.13^c^	1.71 ± 0.81^ab^
IC-Ss	61.5 ± 8.44^bc^	83.5 ± 2.91^ab^	68.1 ± 10.5^ab^	63.1 ± 6.95^bc^	32.7 ± 8.63^ cd^	34.5 ± 10.9^ cd^
IC-R_W_	85.7 ± 10.5^abcd^	98.6 ± 9.94^a^	59.2 ± 12.1^bc^	89.1 ± 6.79^ab^	20.8 ± 2.07^e^	26.5 ± 9.58^cde^
ET-Ss	88.87 ± 4.5^bc^	96.41 ± 14.0^a^	49.20 ± 7.10^c^	79.01 ± 14.9^bc^	59.88 ± 6.58^bc^	48.71 ± 14.4^c^
ET-R_W_	61.50 ± 7.24^bc^	63.86 ± 5.58^bc^	65.13 ± 14.7^bc^	119.37 ± 24.2^a^	62.38 ± 14^bc^	53.58 ± 3.61^c^

IR = inherent resistance, IX_C_ = inherent capacitive reactance, IXL = inherent inductive reactance, IZ = inherent impedance, IC = inherent capacitance, ET = effective thickness, values indicate the mean ± SE, n = 8. The small letter indicates significant difference at (p < 0.05) using Duncan’s multiple range tests.

### 3.4. *Intracellular water utilization parameters of* A. corniculatum

The result of water utilization parameters of *A. corniculatum* is shown in Table 3. Based on IR, IX_C_, IXL, IZ, and IC equations, the IWHC, IWUE, IWHT were obtained, which show the water status in mangrove leaves. The results of IWHC show significant change in low-high salt level, while IWUE and IWHT show nonsignificant results in the S_S_ phase.There is not any prominent change were observed in IWHT when increasing the salt level. Addition of SNP improves the IWHT at low salt level, while nonsignificant results were seen for IWHC and IWUE in the S_S_ phase with SNP application. These results indicates that SNP reflected only IWHT at low salt level. R_W_ shows positive impact on IWHC at H-M level and IWUE at M-L salt level.

**Table 3.** The water utilization parameters of *A. corniculatum* in salt stress and rewatering phase

Salt concentrations	Low (NaCl)	Low (SNP + NaCl)	Medium/medium-low (NaCl)	Medium/medium-low (SNP + NaCl)	High/high-medium (NaCl)	High/high-medium (SNP +NaCl)
IWHC-Ss	440.8 ± 43.6 ^cd^	667.1 ± 102.4^b^	480.7 ± 56.9 ^cd^	520.3 ± 84.2^bc^	131.1 ± 11.6^e^	276.7 ± 15.6^de^
IWHC-R_W_	544.7 ± 92.3^bc^	1112 ± 45.2^a^	402.7 ± 71.2 ^cd^	495.4 ± 46.8^bc^	147.4 ± 41.5^e^	384.3 ± 102 ^cd^
IWUE-Ss	0.16 ± 0.02^bc^	0.07 ± 0.01^c^	0.12 ± 0.18^c^	0.12 ± 0.17^c^	0.21 ± 0.03^bc^	0.10 ± 0.01^c^
IWUE-R_W_	0.15 ± 0.63^bc^	0.05 ± 0.01^c^	0.34 ± 0.09^a^	0.08 ± 0.01^c^	0.31 ± 0.09^ab^	0.16 ± 0.03^bc^
IWHT-Ss	51.1 ± 3.54^a^	48.1 ± 1.13^b^	51.6 ± 0.10^a^	50.1 ± 0.53^a^	52.8 ± 0.07^a^	50.9 ± 0.79^a^
IWHT-R_W_	42.5 ± 1.71^c^	47.6 ± 1.85^ab^	51.1 ± 0.22^a^	45.1 ± 1.79^ab^	49.6 ± 2.83^ab^	52.4 ± 0.09^a^

IWHC = Intracellular water holding capacity, IWUE = water-use efficiency, and IWHT = water-holding time of *A. corniculatum* species, values indicate the mean ± SE, n = 8. The small letter indicates significant difference at (p < 0.05) using Duncan’s multiple range tests.

### 3.5. *Intracellular water utilization parameters of* K. obovate *species*

The results IWHC, IWUE, and IWHT of *K. obovate* species are shown in Table 4. In the S_S_ phase, IWHC and IWHT values were decreased with increasing the salt level, an obvious effect was observed, while IWUE having nonsignificant results were observed during S_S_ phase. The addition of SNP shows clearly the changes on IWHT, but IWHC and IWUE shows nonsignificant results with SNP application in the S_S_ phase. R_W_ technique shows positive impact on IWHC and IWHT at M-L level, and IWUE show nonsignificant results in all salt level with and without SNP application. These results indicate that SNP and R_W_ show different impact on *K. obovate* species in different salt level.

**Table 4.** The water utilization parameters of *K. obovate* species in salt stress and rewatering phase

Salt concentrations	Low (NaCl)	Low (SNP + NaCl)	Medium/medium-low (NaCl)	Medium/medium-low (SNP + NaCl)	High/high-medium (NaCl)	High/high-medium (SNP +NaCl)
IWHC-Ss	1336.4 ± 67.2^bc^	1618.4 ± 40.2^b^	821.5 ± 54.3^cd^	1452.5 ± 82.7^bc^	411.2 ± 85.4^d^	413.7 ± 223.1^d^
IWHC-R_W_	1253.5 ± 114^bc^	1663.6 ± 133^b^	996.2 ± 407^bc^	1870.1 ± 151^a^	868.3 ± 386 ^cd^	326.7 ± 157^d^
IWUE-Ss	0.21 ± 0.05^b^	0.12 ± 0.02^b^	0.09 ± 0.01^b^	0.17 ± 0.06^b^	0.31 ± 0.06^ab^	0.32 ± 0.15^ab^
IWUE-R_W_	0.08 ± 0.01^b^	0.06 ± 0.01^b^	0.14 ± 0.02^b^	0.15 ± 0.02^b^	0.16 ± 0.07^b^	0.72 ± 0.43^a^
IWHT-Ss	49.8 ± 0.94^ab^	37.8 ± 5.08 ^cd^	39.4 ± 2.16 ^cd^	33.6 ± 5.33^de^	24.5 ± 0.23^e^	39.1 ± 0.55^de^
IWHT-R_W_	40.4 ± 5.50 ^cd^	40.5 ± 3.15 ^cd^	44.6 ± 1.04^abc^	47.1 ± 1.35^abc^	26.5 ± 2.54^e^	30.6 ± 0.55^de^

IWHC = Intracellular water holding capacity, IWUE = water-use efficiency, and IWHT = water-holding time of *K. obovate* species, values indicate the mean ± SE, n = 8. The small letter indicates significant difference at (p < 0.05) using Duncan’s multiple range tests.

### 3.6. *Salt transport parameters of* A. corniculatum and K. obovate *species*

Results of salt transport parameters of *A. corniculatum* species are shown in Table 5. Based on the mechanical equations, the USF, STR, and STC were monitored. Results of S_S_ phase show that USF has nonsignificant result in low and medium salt level, but at high salt level, values were increased, while STR values were decreased and increased with increasing salt concentration in low–high salt level. Furthermore, STC has nonsignificant result even in high salt environment. These results display that STC does not reflect in S_S_ phase. Moreover, SNP improves the USF and STR in low and medium salt level, but in higher salt level, SNP is not reflected by USF and STR in the S_S_ phase. When the salt concentration decreased in rewatering phase, nonsignificant results were observed; it means rewatering technique did not reflect the USF, STR, and STC of *A. corniculatum* species.

**Table 5.** Salt transport parameters of *A. corniculatum* species in salt stress and rewatering phase

Salt concentrations	Low (NaCl)	Low (SNP + NaCl)	Medium/medium-low (NaCl)	Medium/medium-low (SNP + NaCl)	High/high-medium (NaCl)	High/high-medium (SNP +NaCl)
USF-Ss	3.51 ± 0.79^c^	3.02 ± 0.24^c^	4.81 ± 0.15^c^	3.75 ± 0.26^c^	10.2 ± 1.19^a^	4.65 ± 0.68^c^
USF-R_W_	2.01 ± 0.67^c^	3.03 ± 0.61^c^	4.21 ± 0.16^c^	2.41 ± 0.25^c^	12.2 ± 4.89^a^	6.42 ± 0.86^bc^
STR-Ss	3.69 ± 0.08^cdef^	7.66 ± 0.82^b^	4.56 ± 0.48^cde^	5.82 ± 0.98^bcd^	1.16 ± 0.16 ^f^	2.94 ± 0.46^def^
STR-R_W_	6.16 ± 1.84^bc^	12.20 ± 1.49^a^	3.49 ± 0.64^cde^	6.14 ± 0.32^bc^	1.86 ± 0.51^ef^	3.45 ± 1.11^def^
STC-Ss	13.1 ± 3.08^b^	23.3 ± 3.78^b^	21.8 ± 1.62^b^	21.3 ± 2.02^b^	11.5 ± 0.45^b^	13.1 ± 0.14^b^
STC-R_W_	12.1 ± 3.27^b^	35.9 ± 4.97^a^	14.7 ± 3.01^b^	14.6 ± 0.95^b^	17.7 ± 1.32^b^	22.6 ± 8.32^b^

USF = Salt flux per unit area, STR = salt transfer rate and STC = salt transport capacity in *A. corniculatum*, values indicate the mean ± SE, n = 8. The small letter indicates significant difference at (p < 0.05) using Duncan’s multiple range tests.

The results of salt transport parameters of *K. obovate* species are shown in Table 6.The results of USF significantly changed in low-high salt level during the S_S_ Phase. STR values were decreased with increasing the salt concentration during the S_S_ phase. SNP application clearly reflected USF at low salt level in the S_S_ phase, but STR and STC shows nonsignificant results with addition of SNP application. R_W_ phase does not improve the USF and STR, but STC was improved specially at the M-L level. These results indicate that SNP and R_W_ both show different impact on salt-resistant parameters of *K. obovate* species in different salt levels.

**Table 6.** Salt transport parameters of *K. obovate* species in salt stress and rewatering phase

Salt concentrations	Low (NaCl)	Low (SNP + NaCl)	Medium/medium-low (NaCl)	Medium/medium-low (SNP + NaCl)	High/high-medium (NaCl)	High/high-medium (SNP +NaCl)
USF-Ss	3.58 ± 0.55^b^	1.79 ± 0.47^c^	1.76 ± 0.16^bc^	1.41 ± 0.29^c^	3.56 ± 0.17^b^	6.31 ± 1.86^a^
USF-R_W_	2.15 ± 0.66^bc^	1.91 ± 0.34^bc^	2.10 ± 0.09^bc^	2.74 ± 0.26^bc^	1.02 ± 0.12^c^	1.25 ± 0.59^c^
STR-Ss	17.8 ± 2.06^ab^	20.8 ± 2.72^ab^	14.5 ± 3.09^bc^	15.1 ± 1.33^bc^	4.45 ± 1.65^c^	4.22 ± 1.81^c^
STR-R_W_	21.7 ± 6.71^ab^	24.8 ± 4.50^a^	10.6 ± 3.55^bc^	18.1 ± 2.55^abc^	5.03 ± 2.98^c^	4.96 ± 2.41^c^
STC-Ss	33.3 ± 3.74^abc^	35.2 ± 5.88^abc^	25.6 ± 6.43^bcd^	21.1 ± 4.05^cde^	15.5 ± 4.46^de^	21.1 ± 6.71^cde^
STC-R_W_	38.7 ± 2.27^ab^	45.2 ± 5.93^a^	22.8 ± 6.5^bcde^	48.4 ± 2.80^a^	18.5 ± 6.26^de^	5.96 ± 2.83^e^

USF = Salt flux per unit area, STR = salt transfer rate and STC = salt transport capacity in *K. obovate* species, values indicate the mean ± SE, n = 8. The small letter indicates significant difference at (p < 0.05) using Duncan’s multiple range tests.

### 3.7. *Water potential (Ψ) of* A. corniculatum *and* K. obovate *species*

**Figure 5.**
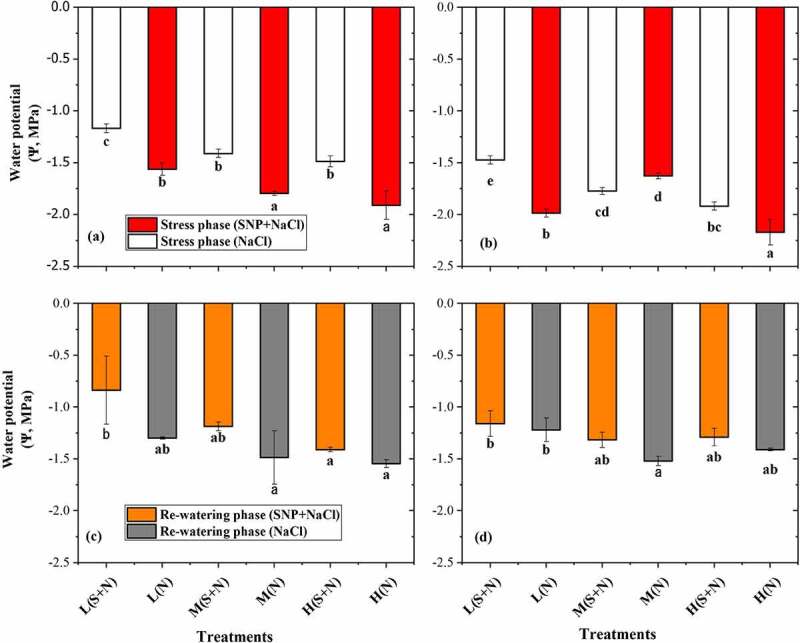
Effect of salt stress and rewatering on water potential (Ψ): (a, c) *A. corniculatum* and (b, d) *K. obovate*. The value n = 12 and small letter indicates significant difference at (*p* < .05) using Duncan’s multiple range tests.

The Ψ of *A. corniculatum* and *K. obovate* species showed significant results in S_S_ and R_W_ phase in Figure 5. In the S_S_ phase, the results were significant with SNP application of *A. corniculatum* species, and it was observed that Ψ was improved by SNP application. During the rewatering phase, Ψ also found higher values as compared to stress phase. Changes were observed in both phase, which indicated that when salt level increased, Ψ of *A. corniculatum* species was decreased. Furthermore, the results of *K. obovate* species also was significant during the stress phase. *K. obovate* obtained higher values in higher salt level, which shows that *K. obovate* are more salt resistant, and the salt storage capacity and vacuole volume are larger. In the R_W_ phase when salt concentration decreased, *K. obovate* species increased the Ψ 34.6%, 6.9%, 48.6%, and 53.9% as compared to stress phase shown in Figure 5d. These results indicate that R_W_ technique and SNP application improve the Ψ.

### 3.8. *Photosynthetic traits of* A. corniculatum *species*

**Figure 6.**
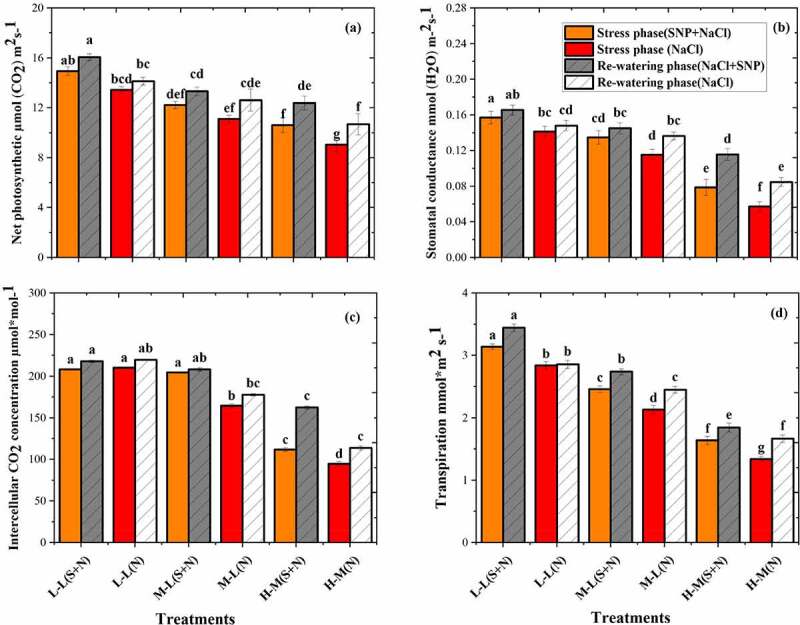
Effect of salt stress and rewatering on (a) net photosynthetic, (b) stomatal conductance, and (c) intercellular CO_2_ (Tr) transpiration of *A. corniculatum*. The value n = 12 and small letter indicates significant difference at (*p* < .05) using Duncan’s multiple range tests.

The photosynthetic parameters of *A. corniculatum* species was significantly decreased in the S_S_ phase and increased in R_W_ phase as shown in Figure 6. The effects of salt varied from time to time, with significant interactions between time and NaCl concentrations. The P_N_ values were decreased by increasing the salt concentration, even at lower concentration. The lowest value was recorded at high salt level and higher value at lower salt level (Figure 6a). Stomatal conductance was a more sensitive parameter in stress condition, as compared to P_N_, and it was noted that gs was significantly decreased in *A. corniculatum* with increasing salt concentration, and more prominent reduction of gs was found in high salt level with and without SNP application inthe stress phase (Figure 6b). Furthermore, similar trend was observed, and the intercellular concentrations CO_2_ (Ci) were also reduced. Thus, the nonsignificant difference was observed in T1, T2, and T3 treatment, although T5 and T6 treatments were sharply decreased. Similarly, the Tr were decreased throughout the stress phase; the important reduction was noted in T5 and T6 treatments. After that, the results of R_W_ phase show that the P_N_ values increased by 14.2% and 15.3%, gs by 47% and 47.3%, Ci by 31.1% and 16.9%, and Tr by 12.6% and 24.3% in T5 and T6 treatments, respectively, compared to stress phase. The reason of increment in physiological parameters during R_W_ phase was the low concentration of NaCl.

### 3.9. *Photosynthetic traits of* K. obovate *species*

**Figure 7.**
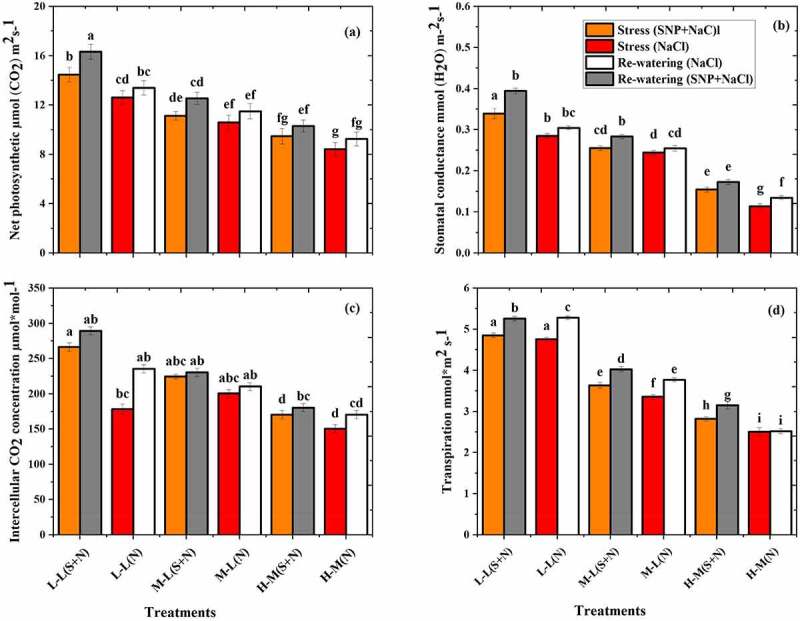
Effect of salt stress and rewatering on (a) net photosynthetic, (b) stomatal conductance, and (c) intercellular CO_2_ (Tr) transpiration of *K. obovate*. The value of n = 12 and small letter indicates significant difference at (*p* < .05) using Duncan’s multiple range tests.

The photosynthetic parameters of *K. obovate* species were significantly decreased in the S_S_ phase and increased in R_W_ phase as shown in Figure 7. The lowest value was recorded at T5 and T6 treatments and higher value at T1 (Figure 7a). It was also noted that T5 and T6 treatments have nonsignificant differences. The gs and Ci obtained higher values in T1 treatment and lower values in T6 treatment. Furthermore, the results of R_W_ phase show that the P_N_ values increased by 8.7% and 9.9%, gs by 10.4% and 15.6%, Ci by 5.7% and 13.3%, and Tr by 11.6% and 0.5% in T5 and T6, respectively, compared to the stress phase.

### 3.10. The correlation between electrophysiological and photosynthetic parameters of mangrove species

Table 7 shows the correlation between electrophysiological, water utilization, salt transport, and photosynthesis parameters of *A. corniculatum* species. During the stress phase, it was observed that USF highly positively correlated with IR, IX_C_, IXL, and IZ and negatively correlated with ET, IWHC, STC, and IWHT. STR was highly positively correlated with IC, IWHC, and gs, while negatively correlation was noted with IR, IX_C_, IXL, and IZ. Furthermore, IWHC significantly correlated with IR, IX_C_, and IZ and positively correlated with the determiner IC, STR, gs, and Tr. Moreover, in the R_W_ phase, IZ with IR, IX_C_, IXL, and USF is positively correlated and IZ with P_N_, gs, Ci, Tr, and Ψ is negatively correlated. USF absolutely correlated with IR, IX_C_, IXL, and IZ, while negatively correlated with IC, ET IWHC, STC, IWUE, IWHT, P_N_, gs, and Ψ. In addition, STC negatively correlated with IR, IX_C_, IXL, IZ, ET, IWHC, USF, IWHT, and gs, while positively correlated with P_N_, Ci, Tr and Ψ.

**Table 7.** Correlation between electrophysiological, water utilization, salt transport, and photosynthesis parameters of *A. corniculatum* species in salt stress and rewatering phase

Salt Stress phase																											
Parameters	IX_C_	IXL	IZ	IC	ET	IWHC		STC		IWUE			IWHT		USF		STR		P_N_		gs		Ci		Tr		Wp
IR	0.97**	0.95**	0.97**	−0.93**	−0.84*	−0.95**		−0.71		0.73			−0.66		0.96**		−0.89*		−0.87*		−0.94**		−0.81		−0.84*		−0.78
IX_C_	.	0.97**	1.00**	−0.93**	−0.81	−0.93**		−0.77		0.75			−0.62		0.96**		−0.88*		−0.76		−0.86*		−0.80		−0.70		−0.73
IXL			0.97**	−0.84*	−0.75	−0.83*		−0.61		0.63			−0.76		0.99**		−0.76		−0.71		−0.80		−0.65		−0.69		−0.70
IZ				−0.94**	−0.81	−0.93**		−0.77		0.75			−0.62		0.96**		−0.88*		−0.76		−0.86*		−0.80		−0.70		−0.74
IC					0.78	0.99**		0.89*		−0.82*			0.38		−0.85*		0.99**		0.83*		0.90*		0.96**		0.73		0.79
ET						0.82*		0.71		−0.80			0.45		−0.73		0.73		0.63		0.80		0.65		0.65		0.43
IWHC								0.86*		−0.86*			0.46		−0.84*		0.97**		0.89*		0.96**		0.93**		0.81*		0.74
STC										−0.90*			0.05		−0.57		0.89*		0.58		0.71		0.90*		0.44		0.51
IWUE													−0.31		0.58		−0.79		0.66		−0.81		−0.76		−0.60		−0.31
IWHT															−0.76		0.26		0.56		0.59		0.15		0.67		0.34
USF																	−0.79		−0.77		−0.83*		−0.68		−0.75		−0.78
STR																			0.84*		0.88*		0.99**		0.72		0.82*
P_N_																					0.96**		0.81		0.97**		0.77
gs																							0.83*		0.94**		0.69
Ci																									0.67		0.80
Tr																											0.67
Rewatering phase																											
IR	0.91*	0.88*	0.95**	−0.76	−0.18		−0.74		−0.10		0.51	−0.87*		0.95**		−0.76		−0.86*		−0.92*		0.19		−0.98**		−0.73	
IX_C_		0.91*	0.99**	−0.90*	−0.14		−0.89*		−0.43		0.24	−0.73		0.90*		−0.88*		−0.94**		−0.95**		0.43		−0.90*		−0.87*	
IXL			0.92**	−0.66	−0.32		−0.66		−0.12		0.09	−0.81		0.98**		−0.62		−0.77		−0.91*		0.09		−0.82*		−0.60	
IZ				−0.87*	−0.11		−0.85*		−0.34		0.29	−0.77		0.93**		−0.86*		−0.93**		−0.94**		0.35		−0.93**		−0.84*	
IC					0.02		0.99**		0.71		−0.36	0.49		−0.65		1.00**		0.96**		0.81		−0.76		0.82*		0.99**	
ET							0.10		−0.09		−0.21	0.44		−0.28		−0.03		0.06		0.35		−0.02		0.21		−0.14	
IWHC									0.74		−0.33	0.45		−0.64		0.99**		0.97**		0.79		−0.78		0.80		0.96**	
STC											0.07	−0.16		−0.01		0.70		0.56		0.29		−0.95**		0.20		0.71	
IWUE												−0.49		0.26		−0.41		−0.35		−0.36		0.24		−0.61		−0.34	
IWHT														−0.87*		0.46		0.55		0.89*		0.06		0.82*		0.44	
USF																−0.63		−0.78		−0.91*		0.03		−0.89*		−0.60	
STR																		0.97**		0.77		−0.76		0.83*		0.98**	
PN																				0.83*		−0.61		0.89*		0.93**	
Gs																						−0.34		0.90*		0.76	
Ci																								−0.33		−0.74	
Tr																										0.78	

** correlation is significant at the 0.01 level, * correlation is significant at the 0.05 level (2-tailed).

Table 8 shows correlation between electrophysiological, water utilization, and salt transport and photosynthesis parameters of *K. obovate* species. In stress phase, IC with IWHC, STC, STR, P_N_, gs, Ci, Tr, and Ψ have positive correlation, while negatively correlated with IR, IX_C_, IXL, and IZ. However, USF was negatively correlated with IC, ET, IWHC, STC, IWUE, IWHT, STR, P_N_ gs, and Ψ and positively correlated with IR IX_C_, IXL, and IZ. Furthermore, IWUE was highly positive correlated with IR, IX_C_, IXL, and IZ, while highly negatively correlated with IC. P_N_ with IC, ET, IWHC, gs, Ci, and Tr have significant correlation, while Ψ has negative correlation with IR, IX_C_, IXL, and IZ and positive correction was observed with IC STC, STR, and Ci. Moreover, in R_W_ phase, IR with IX_C_, IXL, IZ, and IWUE have strongly positive correlation, while it has negative correlation with IC, ET, IWHC, USF, STR, P_N_, gs, Ci, Tr, and Ψ. Furthermore, USF has negative correlation with IC, ET, IWHC, STC, P_N_, gs, Tr, and Ψ. However, IWHT is negatively correlated with IR, IX_C_, IXL, IZ, STC, and IWUE. Additionally, IC with P_N_, gs, Ci, Tr, and Ψ have significant correlation, and P_N_ with its gs, Ci, Tr, and Ψ have positive correlation.

**Table 8.** Correlation between electrophysiological, water utilization, salt transport, and photosynthesis parameters of *K. obovate* species in salt stress and rewatering phase

Salt Stress phase																
Parameters	IX_C_	IXL	IZ	IC	ET	IWHC	STC	IWUE	IWHT	USF	STR	P_N_	gs	Ci	Tr	Wp
IR	0.81*	0.78	0.86*	−0.88*	−0.39	−0.75	−0.95**	0.90*	−0.32	0.77	−0.92*	−0.67	−0.81*	−0.87*	−0.57	−0.90*
IX_C_		0.97**	0.99**	−0.95**	−0.66	−0.87*	−0.77	0.92**	−0.35	0.57	−0.78	−0.79	−0.90*	−0.75	−0.78	−0.69
IXL			0.98**	−0.87*	−0.57	−0.79	−0.78	0.90*	−0.13	0.39	−0.71	−0.64	−0.77	−0.70	−0.62	−0.62
IZ				−0.93**	−0.61	−0.86*	−0.83*	0.93**	−0.26	0.56	−0.80	−0.73	−0.86*	−0.78	−0.71	−0.71
IC					0.65	0.87*	0.85*	−0.93**	0.41	−0.71	0.91*	0.89*	0.96**	0.88*	0.83*	0.86*
ET						0.89*	0.31	−0.36	0.31	−0.49	0.63	0.85*	0.77	0.62	0.88*	0.31
IWHC							0.65	−0.69	0.41	−0.73	0.85*	0.90*	0.93**	0.82*	0.89*	0.61
STC								−0.90*	0.05	−0.57	0.89*	0.59	0.71	0.90*	0.44	0.94**
IWUE									−0.31	0.58	−0.79	−0.66	−0.81	−0.76	−0.60	−0.84*
IWHT										−0.76	0.29	0.57	0.59	0.15	0.67	0.25
USF											−0.79	−0.77	−0.82*	−0.68	−0.75	−0.69
STR												0.85*	0.89*	0.99**	0.72	0.91*
P_N_													0.97**	0.82*	0.97**	0.70
gs														0.84*	0.94**	0.76
Ci															0.67	0.91*
Tr																0.53
Rewatering phase																
IR	0.92**	0.94**	0.98**	−0.45	−0.22	−0.68	−0.07	0.94**	−0.20	−0.01	−0.37	−0.40	−0.31	−0.42	−0.27	−0.08
IX_C_		0.99**	0.98**	−0.69	−0.42	−0.84*	0.08	0.99**	−0.55	0.32	−0.45	−0.50	−0.60	−0.65	−0.52	−0.08
IXL			0.97**	−0.61	−0.37	−0.79	0.08	0.99**	−0.51	0.24	−0.38	−0.42	−0.54	−0.58	−0.44	0.03
IZ				−0.58	−0.35	−0.79	0.03	0.97**	−0.37	0.16	−0.41	−0.46	−0.46	−0.55	−0.39	−0.12
IC					0.45	0.87*	0.18	−0.63	0.82*	−0.87*	0.83*	0.88*	0.97**	0.99**	0.96**	0.53
ET						0.71	−0.28	−0.29	0.61	−0.36	0.13	0.14	0.43	0.35	0.26	−0.03
IWHC							0.12	−0.76	0.73	−0.58	0.68	0.71	0.79	0.82*	0.71	0.35
STC								0.07	−0.16	−0.01	0.70	0.57	0.11	0.24	0.25	0.54
IWUE									−0.49	0.26	−0.41	−0.46	−0.55	−0.60	−0.48	−0.02
IWHT										−0.87*	0.46	0.51	0.91*	0.80	0.80	0.03
USF											−0.63	−0.70	−0.94**	−0.89*	−0.93**	−0.43
STR												0.98**	0.75	0.86*	0.84*	0.76
P_N_													0.81	0.91*	0.89*	0.79
gs														0.97**	0.97**	0.39
Ci															0.98**	0.56
Tr																0.55

** correlation is significant at the 0.01 level, * correlation is significant at the 0.05 level (2-tailed).

### 3.11. Different salt-resistant capacities of mangrove species

As shown in Table 9, the salt outflow capacity is defined as C1 = 0.25 (IR_N_+ IXC_N_ +IXL_N_+ IZ_N_), salt dilution capacity as C2 = 0.25 (IC_N_+ET_N_+IWHC_N_+STC_N_), ultrafiltration capacity as C3 = 0.25 (IWUE_N_+IWHT_N_+USF_N_+STR_N_), and total salt resistance capacity as T = (C1+C2+C3) /3 in salt-stress and rewatering phase of *A. corniculatum* species. Hence, in Table 9, we assess the combined effects of three different capacities in diverse salt levels with SNP application. In both phases, it was noted that the SNP application shows obvious effects as compared to where species received only NaCl treatment. In C1 capacity, *A. corniculatum* species obtained higher values of 0.88 in S_S_ phase (SNP + NaCl) and 0.79 in R_W_ phase. In C2 capacity, higher values were noted with SNP application: 0.62, 0.46, and 0.37, while in the C3 capacity, higher values were observed, 0.66, 0.60 and 0.20, correspondingly as compared to the stress phase. Therefore, it was state that SNP application was more reflected at low salt level in these different capacities. Moreover, R_W_ does not improve these capacities in high salt level because *A. corniculatum* cannot tolerate high salt, but in the same salt level, SNP shows better results.

**Table 9.** Salt outflow capacity (C1), salt dilution capacity (C2), and salt ultrafiltration capacity (C3) of *A. corniculatum* in salt-stress and rewatering phase

Different salt capacities	Low (NaCl)	Low (SNP + NaCl)	Medium/medium-low (NaCl)	Medium/medium-low (SNP + NaCl)	High/high-medium (NaCl)	High/high-medium (SNP +NaCl)
*C1-Ss*	0.50	0.88	0.46	0.64	0.04	0.31
*C1-R_W_*	0.52	0.79	0.22	0.49	0.07	0.18
*C2-Ss*	0.41	0.62	0.40	0.46	0.18	0.37
*C2-R_W_*	0.44	0.69	0.35	0.49	0.26	0.38
*C3-Ss*	0.34	0.66	0.50	0.60	0.05	0.20
*C3-R_W_*	0.31	0.71	0.40	0.30	0.14	0.29
*T-Ss*	0.42	0.72	0.45	0.57	0.09	0.30
*T-R_W_*	0.42	0.73	0.32	0.43	0.16	0.28

Table 10 shows three different capacities of *K. obovate* species in S_S_ and R_W_ phase. In stress phase C1 and C3 capacity, higher values were obtained only in low salt level with SNP application, 0.80 and 0.84 respectively, but in moderate and high salt levels, SNP does not improve the C1 and C3 capacities of *K. obovate* species; these results indicate that SNP cannot conducive the adaptability of *K. obovate* species in high salt level. However, C2 capacity results are almost same at low and moderate salt level, but SNP helps to improve the C3 capacity of *K. obovate* species in low sat level. Furthermore, R_W_ technique is beneficial for *K. obovate* species, and when the salt level decreased from M-L in C3 capacity, there was great improvement as compared to NaCl, and also at H-M level, R_W_ shows good impact as compared to S_S_ phase. SNP application and R_W_ technique have diverse effects on C1, C2, and C3 capacities of *K. obovate* species.

**Table 10.** Salt outflow capacity (C1), salt dilution capacity (C2), and salt ultrafiltration capacity (C3) of *K. obovate* species in salt stress and rewatering phase

Different salt capacities	Low (NaCl)	Low (SNP + NaCl)	Medium/medium-low (NaCl)	Medium/medium-low (SNP + NaCl)	High/high-medium (NaCl)	High/high-medium (SNP +NaCl)
*C1-Ss*	0.38	0.80	0.60	0.65	0.18	0.14
*C1-R_W_*	0.50	0.63	0.32	0.46	0.50	0.15
*C2-Ss*	0.44	0.58	0.58	0.50	0.34	0.35
*C2-R_W_*	0.53	0.58	0.39	0.44	0.46	0.27
*C3-Ss*	0.63	0.84	0.51	0.53	0.25	0.25
*C3-R_W_*	0.55	0.65	0.35	0.74	0.33	0.11
*T-Ss*	0.48	0.74	0.56	0.56	0.26	0.25
*T-R_W_*	0.53	0.62	0.36	0.54	0.43	0.17

## 4. Discussion

The current study measures electrophysiological parameters of *A. corniculatum* and *K. obovate* species in S_S_ and R_W_ phase. The electrophysiological parameters associated with plant cell, vacuole volume, and almost all life activities, if plant is stressed, practically all life activities are impacted. Nitric oxide (NO) application could improve the plant stress resistance and effects on photosynthesis system, antioxidant enzyme system, and metabolism system.^32^ The cell can be considered as a concentric sphere capacitor by their resistor inductor functions,^2,5^ in which the electrolytes of a capacitor are equivalent to electric ions, ion groups, and dipoles. When the cell membrane permeability changes were observed, at that time plant leaf is subjected to a C_F_ stimulus.

Consequently, electrolyte concentrations in inner and outer side changes in the cell membrane, which leads to variation in the leaf C, R, and Z. The Nernst equation could be utilized to expose the electrical potential formed by ions and used to quantitatively describe the diffusion gradient of electrolytes in the outer and inner side of cell membrane.^33^ The Nernst equation, Z (or X_C_) $p_{o}+k_{2}\;e^{-b_{2F}}$, and theoretical intrinsic relationships between the CF and Z and XC of mangroves species was exposed in this paper. The process in which the decreased internal energy of the system can be change into the work done by the system is a concept of Gibbs free energy. According to Gibbs free energy, C = x + KF of the theoretical inherent relationships between the leaf C_F_ and C. The results demonstrated that the fitting equations of R-C_F_, X_C_–C_F_, XL-C_F_, Z-C_F_, and C–C_F_ have positive correlations, which highlighted the existence of the above-mentioned inherent mechanism. In the current study, the IR, IX_C_, IXL, IZ, IC, and ET values of inherent electrical parameters in mangrove leaves were successfully obtained with the help of inherent mechanical relationships between the C_F_ and leaf Z, X_C_, XL, R, and C.

The nonsignificant results of IR, IX_C_, and IXL of *A. corniculatum* species were observed in low and moderate salt levels. IC results had significant change when the salt concentration was increased. SNP application shows positive impact on IX_C_ and IC in different salt levels. These results indicate that IX_C_ and IC are reflected by SNP in long-term and low salt environment, while ET shows positive impact at medium salt level when receiving the SNP application. The ET in plant leaf is highly variable, which depends on plant species.^2^ These results correctly clarify the phenomenon of life in plant: when the concentration of electrolyte is low and intracellular water in plant leaves is sufficient, then plant contains low IR, IZ, IX_C_, and IC and ET. Moreover, the R_W_ technique does not help to improve these electrical parameters expect ET at moderate salt level. Furthermore, IR IX_C_, IXL, and IZ parameters state the salt outflow capacity in plant.

The *K. obovate* species having nonsignificant results were noted of IR, IX_C_, and IXL, while IC and ET have significant results. These results demonstrate that IC and ET are clearly reflecting the *K. obovate* species in stress phase. In our previous study, we noted that the vacuole volume of *K. obovate* species was higher, which enables it to store more salt even in high salt level.^34^ The *K. obovate* species grown under great water state had higher IWHC, which indicate and support the higher IC and ET values of *K. obovate* species. Excluding the IC and ET, the results of remaining parameters were nonsignificant in rewatering, while the IC and ET were considerably improved at the M-L salt level.

The IWHC, IWUE, and IWHT associated with plant cell volume and water utilization parameters. These parameters display water status in plant leaves, representing the ion concentration and intercellular water occupied in plant cell. The results of IWHC in stress phase was significant and reflect of *A. corniculatum* species, while IWUE has nonsignificant results during the S_S_ phase. The results also indicates that SNP application and rewatering technique show positive impacts on IWHT, which improve by SNP in low salt level, while IWUE show better results at moderate salt level with SNP application. Moreover, the results of *K. obovate* species of IWHC and IWHT were significant and IWUE was nonsignificant, which shows that IWHC and IWHT reflect the *K. obovate* species in stress phase when the salt level increased. Because *K. obovate* species have bigger vacuole volume, it keeps lower solute concentration in vacuole;^34^ hence, the water status of the plant leaf was unaffected. A recent research study reported that with the help of electrophysiological parameter, the metabolism of water status of various plant was evaluated.^2^

Moreover, in the mesophyll cells, organelles and cell are existing enclosed within the cell membrane composed of 40% proteins, 50% lipids, and 2 to 10% sugars.^35^ Inductive reactance and inductance are affected by binding protein and capacitive reactance and capacitance by the surface proteins. Thus, the mesophyll cell can be regarded as a concentric sphere capacitor with both inductor and resistor function, and the ions, ion groups, and electric dipoles are equivalent to the electrolytes of a capacitor.^35^ Previously, the monitoring of the salt transport capacity of plants has hardly been described. In the current study, the salt transport capacity of mangroves species based on mechanical equation of IR, IX_C_, IXL, IZ, and IC was obtained. The results were significant of STR in both phases (S_S_ and R_W_) of *A. corniculatum* species, while STC have nonsignificant results. USF was reflected by *A. corniculatum* species only at higher salt level. These result shows that STR was reflected by *A. corniculatum* species in both phases. Furthermore, SNP application could improve the USF and STR in low and medium salt level, but in the high salt level, USF and STR were not reflected in the S_S_ phase. However, rewatering does not improve the salt transport capacity of *A. corniculatum* species.

Moreover, USF, STR, and STC were reflected in stress phase of *K. obovate* species, and obvious changes were observed when salt concentration increased. In our recent study, we know that v =$\alpha \sqrt{CP^{3}}$, V = volume of vacuole, and CP = physiological capacitance if volume of vacuole is bigger; thus, physiological capacitance was also higher.^34^ The SNP application also improved and reflected the USF during the S_S_ phase, and rewatering technique also improve the USF and STC. These results indicate that as compared to SNP, rewatering does not improve and reflect the STR.

The variation in Ψ of both mangrove species are shown in Figure 5. The Ψ decreased, which indicated that solute concentration inside the cell could have a threshold value for dysfunction of cells and increased the Ψ of plant. Present results state that when salt concentration increased, Ψ also decreased, and it means solute concentration inside the cell was higher and inhibited the leaf cell. The cell volume depends on salt storage capacity. During the R_W_ phase, Ψ shows good response as mentioned in Figure 5c,d, because solute concentration inside the cell was decreased, so that the Ψ also decreased. Additionally, physiological parameters were more inhibited of *A. corniculatum* species in S_S_ phase. Presence of Na^+^ can be harmful for plant metabolism and potentially kill the plant.^36^ The most common explanation for Na^+^ toxicity is that it has an inhibitory effect on the activities of enzymes. Therefore, SNP also increase the leaf cell size and helps to upstand in stress condition.^37^ When the leaf cell size increases, it directly impacts on salt secretion rate of *A. corniculatum*. The P_N_ significantly decreased with increasing the salt concentration, but in those treatments where plant received SNP + NaCl application, the P_N_ values were observed higher than other treatments. Furthermore, other photosynthesis-related parameters such as gs and Tr also decreased with increasing salt concentration.^38^ When the salt entered in vacuole, it impacts on whole cell especially solute concentration in the cytosol. Generally, salt glands in recretohalophytes lack a big central vacuole but contain others microvacuoles; these microvacuoles may have a connection with active metabolism of salt glands.^39^ Moreover, the application of rewatering demonstrations better development of *A. corniculatum* and obtained higher values in all treatments of photosynthetic parameters, which is exposed in Figure 6. The increment is noticed in all treatment, but more dominated values were seen in where mangrove received high salt concentration. The P_N_ values increased by 14.2% and 15.3% and gs by 47% and 47.3% in T5 and T6 treatments compared to stress phase. Because when the salt concentration decreased, at that time leaf cell maintained their metabolism and water status, which helps to increase P_N_ values and gs. Present results supported by findings of Azeem et al. and Javed et al.^40,41^ reported that application of R_W_ helps the plant to maintain their development, and the effects of salt stress could be reduced by R_W_.

The physiological parameter of *K. obovate* species decreased by NaCl in the S_S_ phase as shown in Figure 6. The reason of reduction in physiological parameters are presence of NaCl in water, which increase the osmotic stress, resulting in rapid closing of gs and decreased the P_N_. The *K. obovate* species contain larger leaf cell size and vacuole volume as compared to *A. corniculatum*. With their particular mechanisms, the vacuole can store salts and exclude them. ^42^ The P_N_ and gs in T1 treatment obtained higher values and lower value in T6. As reported by Xu et al.,^11^ the high salinity inhibited photosynthesis by closing of gs, and on the other hand, the reduction of CO_2_ assimilation was the main cause of salt stress on photosynthetic activity. The CO_2_ assimilation rate and gs both are decreased when increasing the environmental salinity.^43^ Furthermore, during the R_W_ phase when salt concentration decreased as compared to S_S_ phase, the photosynthetic parameters show higher value than stress phase (Figure 6). Javed et al.^41^ reported that gs was sharply responsive when there was changes in the soil water salinity. Similar trend was seen in the present study when decreasing the salt concentration, mangrove shows higher value of gs and other related parameters in the R_W_ phase. According to the results, we observed that the electrical parameters better characterized the response of mangroves in stress condition as compared to photosynthesis parameters. Finally, in this study, intracellular water-use indices IWHC, IWUE, and IWHT are based on plant inherent electrical parameters and have ability to monitoring the intracellular water metabolism in mangroves and IR, IX_C_, and IXL of plants electrophysiological information, which could efficiently manifest the composition and salt transport characteristics of membrane protein in mangroves.

Furthermore, in Table 7, it was demonstrated that USF highly positively correlated with IR, IX_C_, IXL, and IZ in both phases (S_S_ and R_W_), while negatively correlated with ET, IWHC, STC, and IWHT of *A. corniculatum* species. STC positively correlated with IC, ET, IWHC, STR P_N_, Ci, Tr, and Ψ in stress phase and negatively correlated with other parameters, but in R_W_ phase, only P_N_, Ci, Tr, and Ψ show positive correlation with STC. Moreover, correlation of IC with IWHC, STC, STR, P_N_, gs, Ci, Tr, and Ψ is positive for *K. obovate* species, whereas USF negatively correlated with IC, ET, IWHC, STC, IWUE, IWHT, STR, P_N_ gs, and Ψ. Furthermore, IWHC has positive correlation with IC and ET because IC and IWHC are directly connect with vacuole volume; thus, when IWHC increased, similarly IC and ET also increased. In our previous study, P_N_ shows positive correlation with gs, Ci, Tr, and Ψ.^34^ Similarity was seen in current study, where P_N_ positively correlated with photosynthesis parameters. In the rewatering phase, IR with IX_C_, IXL, IZ, and IWUE have strongly positive correlation and IC with P_N_, gs, Ci, Tr, and Ψ have significant correlation of *K. obovate* species.

**Figure 8.**
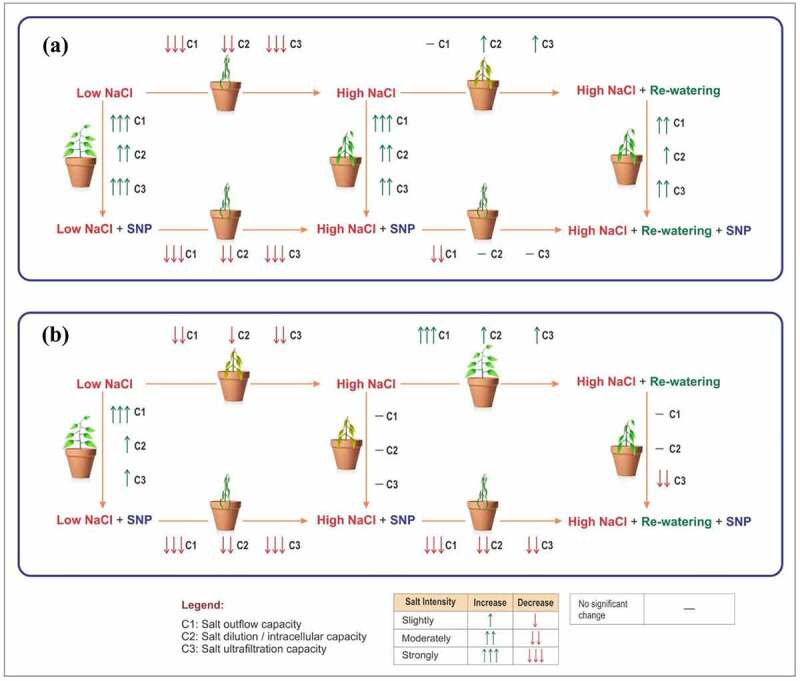
Hypothetical model of mangrove species in different salt level with influences of SNP and rewatering: (a) *A. corniculatum* and (b) *K. obovate* species.

Furthermore, the results of three different capacities are shown in Tables 9 and 10 of *A. corniculatum* and *K. obovate* species. Salt outflow capacity, salt dilution capacity, and salt ultrafiltration capacity of *A. corniculatum* species were improved with SNP application in all salt levels. At the low salt level, by SNP application, *A. corniculatum* species obtained higher value of 0.88, in medium salt level, 0.64, and high level, 0.31; when the salt level increased, simultaneously the values of C1 capacity were decreased. Because *A. corniculatum* species cannot be tolerant in the high stress environment, but SNP shows improvement only in C2 and C3 capacities when salt level reach at 400 mM (Figure 8a). R_W_ technique shows positive impact and helps to improve the only ultrafiltration capacity of *A. corniculatum* species.

Moreover, C1 and C3 capacities of *K. obovate* species were improved by SNP application of *K. obovate* species in low salt level, but in moderate and high salt level, SNP did not improve the C1 capacity. These results indicate that SNP application improves these capacities at low salt level but not beneficial for the adaptability of *K. obovate*. While, during the low salt level, C3 capacity obtain higher values 0.84 by *K. obovate*. R_W_ technique improve all capacities and C3 capacity at M-L level was greatly improved. The SNP application used as supplementary and in low salt level shows obvious impact on both species. But at high salt level, SNP shows nonsignificant results of *K. obovate* species. These results display that electrophysiological parameter could be used to characterize the differences in both mangrove species and clearly reflecting C1, C2, and C3 capacities in different salt levels. These parameters give positive, reliable, and quick results as compared to photosynthesis parameters. Previous research studies reported that the SNP is helpful for restoration of plant in the stress environment.^44–46^.

Figure 8 demonstrates that the salt tolerance mechanism of both species with impacts of SNP and R_W_ technique. The SNP application at low salt level increases the C1, C2, and C3 capacity of both species, but at high salt level, SNP cannot help to improve the salt-resistance capacities, which indicate that high concentration of salt stress is not accompanied by the peroxidation stress of membrane lipid, while SNP improves the antioxidant capacity. Moreover, R_W_ helps to effectively improve salt tolerance of *K. obovate* under high salt concentrations, which indicates that *K. obovate* species is more salt resistant because this process is consistent with the actual situation; under high salt, *A. corniculatum* is irreversible and R_W_ cannot reverse the salt resistance of *A. corniculatum* species.

## 5. Conclusion

The present study initially verifies the relationship of fitting equations between the electrophysical parameters, including R, XC, XL, Z, and C, and the CF of two different mangrove species in the Ss and R_W_ phases. These inherent electrical parameters IR, IX_C_, IXL, IZ, and IC of mangrove leaves were detected for the first time with the help of mechanical equations. Subsequently, the IWHC, IWUE, and IWHT of the intracellular water-use parameters in the mangroves were defined based on the inherent electrical parameters of the water status of metabolism. These parameters accurately exposed the life activities and changes in water status in mangrove species and could be hypothetically used for the intercellular metabolism water status in mangroves. The salt transport indices USF, STR, and STC were also monitored based on IR, IX_C_ IXL, IZ, and IC. The results show that the electrical parameters IC and ET better reflect the *A. corniculatum* species, and the IWHC also shows significant results under stress conditions, while *K. obovate* species is more reflected by IR, while IZ and other electrical parameters show nonsignificant results. Furthermore, the IWHC, IWHT, USF, STR, and NTC could better reflect under different salt levels of *K. obovate* species. Three different capacities of *A. corniculatum* species were reflected by SNP application at all salt levels, especially at low salt levels. These capacities were greatly improved by SNP, while obvious changes in *K. obovate* species were observed at low salt levels. Under low concentration SNP, can effectively improve the salt tolerance of both mangrove species. However, under high concentration, SNP can effectively improve the salt tolerance of *A. corniculatum*. Moreover, under high concentration, R_W_ only effectively improves the salt resistance of *K. obovate*, which indicates that *K. obovate* is more salt-resistant because R_W_ process is consistent with the actual situation, the response of *A. corniculatum* to high salt stress is irreversible, and R_W_ cannot reverse the salt resistance of *A. corniculatum* species. Therefore, it is concluded that these three capacities help to understand salt tolerance mechanism and different adaptive strategies of both mangrove species in diverse salt levels.
